# Prediction of Anemia in Adenomyosis Patients Using Transvaginal Ultrasound Radiomics

**DOI:** 10.3390/diagnostics16172827

**Published:** 2026-09-02

**Authors:** Chaeheon Lee, Young Jae Kim, Han-song Song, Soohyun Oh, Kwang Gi Kim

**Affiliations:** 1 KMAIN Co., Ltd., 621-622, 54 Chang-eop-ro, Sujeong-gu, Seongnam-si 13355, Republic of Korea; bethelight@g.skku.edu; 2 Gachon Biomedical & Convergence Institute, Gachon University Gil Medical Center, Incheon 21565, Republic of Korea; kimyj10528@gmail.com; 3 Department of Obstetrics and Gynecology, Gachon University Gil Medical Center, Incheon 21565, Republic of Korea; evehansong@gmail.com; 4 Department of Biomedical Engineering, Gachon University Collage of Medicine, Incheon 21565, Republic of Korea

**Keywords:** adenomyosis, anemia, transvaginal ultrasound, radiomics, machine learning

## Abstract

**Background:** Abnormal uterine bleeding (AUB) caused by adenomyosis can result in significant iron-deficiency anemia. Given that transvaginal ultrasound (TVUS) is commonly performed during routine examinations, its assessment may facilitate preemptive treatment. **Methods:** In this study, TVUS images from patients with surgically confirmed adenomyosis were preprocessed to minimize intra- and inter-scan variability. Radiomics features were extracted using PyRadiomics to train automated machine-learning classifiers under k-fold nested cross-validation. Multiple dataset configurations were evaluated, including feature extraction from a custom region-of-interest (ROI) of the uterine corpus, whole-image features adjusted for uterine size, and supplementary variables from complete blood counts (CBC). **Results:** Radiomics-centered models demonstrated modest discrimination (mean accuracy 0.606–0.618). While incorporating CBC variables with radiomics features substantially improved performance compared to radiomics alone, models trained exclusively on CBC data yielded overall higher results. **Conclusions:** These findings indicate that quantitative features derived from routine TVUS provide modest complementary information for predicting anemia. While they do not offer clear incremental value when highly discriminatory clinical data (CBC) are available, TVUS radiomics may serve as a supplementary diagnostic tool in settings where blood tests are incomplete or unavailable. Further validation in larger, multi-institutional cohorts with patient-level separation is warranted.

## 1. Introduction

Adenomyosis is a common benign gynecologic condition characterized by the infiltration of endometrial glands and stroma into the myometrium, resulting in myometrial hypertrophy and uterine enlargement. Clinically, adenomyosis is often associated with heavy or prolonged menstrual bleeding, as well as symptoms such as dysmenorrhea, dyspareunia, and chronic pelvic pain [1]. It is specifically listed as a cause of abnormal uterine bleeding (AUB) in the PALM-COEIN classification system, and excessive AUB can lead to chronic iron-deficiency anemia, which can significantly impair quality of life [2]. In some cases, bleeding may progress to severe anemia, necessitating emergency department visits, blood transfusions, or surgical intervention [3,4]. These potential consequences highlight the clinical importance of early identification of patients at risk for anemia.

Despite the clinical burden of anemia in adenomyosis, predicting which patients will develop severe anemia remains challenging. Approximately 30% of women with adenomyosis are reported to be asymptomatic [5,6], and in routine practice, hemoglobin decline is often recognized only after substantial bleeding has occurred. Transvaginal ultrasonography (TVUS) is the recommended first-line imaging modality for suspected adenomyosis, providing detailed information on uterine morphology [7]. Ultrasonographic features such as uterine enlargement, asymmetric myometrial thickening, myometrial cysts, and heterogeneous echotexture—systematically described in the Morphological Uterus Sonographic Assessment (MUSA) criteria—have been shown to correlate with disease extent and symptoms such as dysmenorrhea [8,9,10].

However, the direct association between these imaging features and anemia has not been well established [11]. Several factors currently limit accurate ultrasonographic evaluation. First, assessment is largely qualitative and subject to interobserver variability, which may reduce its ability to capture complex structural patterns related to disease severity [12]. Furthermore, the criteria described above are macroscopic; smaller-scale heterogeneity may need to be considered alongside these well-known sonographic features to establish a connection with specific outcomes, such as anemia.

Recent advances in quantitative image analysis have enabled the extraction of reproducible features from routine medical images, providing a more objective evaluation of tissue characteristics [13]. Radiomics is one such approach [14,15,16]. This method involves high-throughput extraction of quantitative features from images, computed from microscopic tissue architecture represented in pixel or voxel distributions, depending on image dimension [15]. Features are categorized into several classes, including shape-based features, such as macroscopic measures of sphericity and size, and texture-based features, defined by spatial relationships between individual pixels [17]. Such features have successfully been utilized in studies to diagnose a wide range of conditions as well as post-operative outcomes [18,19]. The extraction process allows for automation: although a specific region or volume of interest (ROI) must be indicated, neural networks such as nnU-Net, short for “no new U-Net”, have demonstrated successful automatic segmentation, suggesting the potential for fully automated workflows [20].

Radiomics-based features have shown value in both diagnostic and prognostic imaging tasks. In lung cancer, recent approaches using machine learning and deep learning have been applied to distinguish benign from malignant lesions and support subtype classification [21,22]. Yan et al. developed a machine-learning model combining preoperative MRI radiomic features with clinical data to predict postoperative pancreatic fistula after pancreaticoduodenectomy. A retrospective cohort of 139 patients was examined, with the integrated radiomics–clinical model outperforming the radiomics-only model and achieving an area under the curve (AUC) of 0.899 in the test set [23].

Machine and deep learning-based applications have also demonstrated promise in obstetric and gynecologic ultrasound. A systematic review by Jost et al. summarized 189 studies of AI-assisted ultrasound in obstetrics and gynecology, including automated plane acquisition and endometrial evaluation [24]. In gynecologic oncology, Christiansen et al. externally validated transformer-based models for ovarian cancer detection using 17,119 ultrasound images from 3652 patients across 20 centers in eight countries. The models outperformed expert and non-expert examiners and reduced simulated referrals to experts by 63% [25]. Together, these studies support the premise that AI-derived ultrasound features can capture clinically meaningful information beyond conventional visual assessment, providing a rationale for radiomics-based prediction using routine transvaginal ultrasound images.

Applying these approaches to ultrasonographic images of adenomyosis may allow a more refined assessment of disease-related structural changes and their clinical consequences. In addition to quantifying tissue heterogeneity, radiomics offers the advantage of ease in data accumulation [17]. A more direct association between morphological features of adenomyosis and anemia may thus be identifiable, particularly when analyzed across a cohort. Importantly, radiomics can extract this structural information using data already obtained from standard clinical care. Prior studies have successfully applied radiomics to MRI images of adenomyosis patients to predict treatment outcomes, demonstrating statistically significant results [26,27]. In particular, Burla et al. showed that radiomic features extracted from T2-weighted MRI could be used to detect adenomyosis in a proof-of-principle, 15-patient cohort, achieving an AUC of 0.78 to 0.98 [28].

Therefore, this study was designed as a proof-of-concept investigation to evaluate the association between preoperative ultrasonographic features of adenomyosis and the presence of anemia in premenopausal patients undergoing hysterectomy. By applying radiomics to analyze both macroscopic and microscopic features from routinely acquired ultrasonographic data, the study aims to provide preliminary evidence of a potential link between adenomyosis morphology and anemia. This association can be further explored in future patient-level validation studies.

## 2. Materials and Methods

### 2.1. Study Cohort

Ultrasound images of premenopausal patients diagnosed with adenomyosis were originally obtained by Gachon University Gil Medical Center between July 2010 and May 2022. In this retrospective observational study, conducted in accordance with the ethical guidelines of the Declaration of Helsinki and approved by the Institutional Review Board (IRB) of Gachon University Gil Hospital (IRB no. GBIRB2026-162), images from a total of 382 patients with an age range of 28–50 years were accessed and reviewed. All patients underwent hysterectomy, and the images used were acquired as part of preoperative procedures. Resected tissue was examined histologically to confirm the diagnosis. Among these patients, 120 were diagnosed with iron-deficiency anemia based on blood tests conducted prior to operation. Anemia was diagnosed based on the WHO definition of a hemoglobin concentration below 12.0 g/dL [29]. TVUS images were acquired using Aplio 500 (Toshiba Medical Systems Corporation, Otawara, Japan), UGEO WS80A (Samsung Medison Co., Ltd., Seoul, Republic of Korea), and Voluson E8 (GE Healthcare Austria GmbH & Co. OG, Zipf, Austria) ultrasound systems.

A general assessment of all images was conducted by an OB/GYN author to ensure that sonographic features of adenomyosis were adequately demonstrated. This was defined as at least one of the three direct signs of adenomyosis as indicated in the MUSA criteria being present alongside one or more indirect features, such as a globular uterus, asymmetrical thickening and fan-shaped shadowing [8].

B-mode greyscale TVUS was selected as the modality of choice, as other imaging modalities, such as color Doppler, were available for only a small subset of patients. Standard data augmentation techniques, such as random flips and rotations, were not applied to preserve clinical plausibility. Patient-level grouping was first tested on a smaller dataset with one image per patient. An expanded dataset containing up to four images per patient was used as the main set, with each image representing a different acquisition angle and treated as a separate sample. This approach was adopted because while each image captured distinct aspects of the uterus, views were not standardized across patients to support multi-branch analysis, as described in [30]. This study was designed as a proof-of-concept investigation of the relationship between sonographic features of adenomyosis and anemia. Consequently, results are preliminary, and the potential for data leakage should be noted.

### 2.2. Dataset Construction

Two primary categories were created from the compiled images. In the first category, a manually defined region of interest (ROI) encompassing the uterine corpus was used to segment the images (referred to as the “uterine-specific” or “specific” ROI). In the second category, segmentation was applied to the entire image (referred to as the “whole-image” ROI).

**Figure 1:**
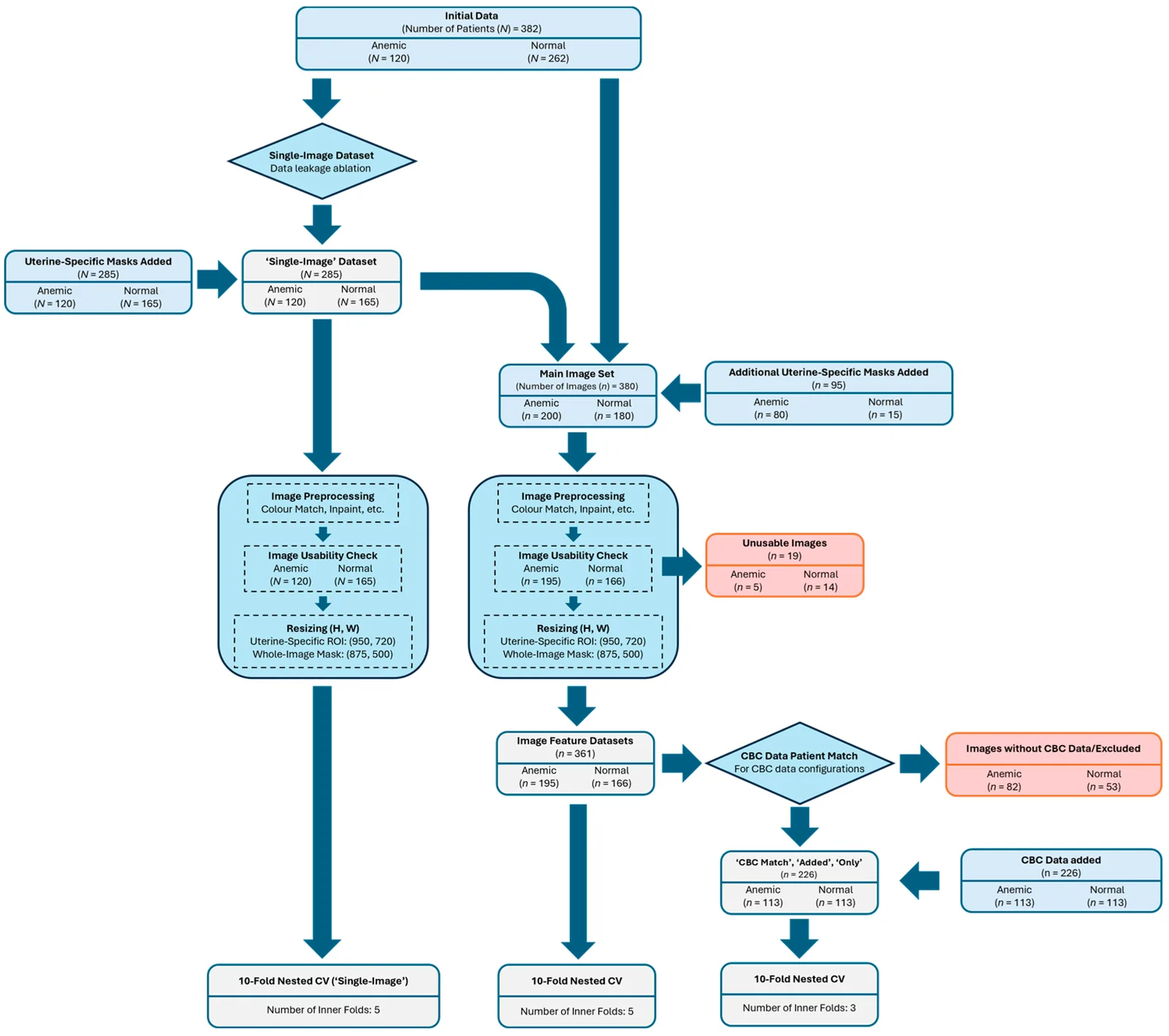
Overall study flow diagram. “*N*” denotes the number of patients, and “*n*” denotes the number of images. Two ROI configurations exist as shown below in Table 1 but are only indicated here when necessary. Light-gray boxes indicate the datasets included in the final analysis and corresponding procedures. Lighter blue boxes indicate image or mask sets added to the workflow, whereas darker blue boxes indicate intermediate processing steps. Dotted boxes are used for substeps in image preparation. Red boxes indicate excluded images or data. Diamonds represent dataset-selection or matching steps, and arrows indicate the direction of the workflow.

Figure 1 shows the original number of patients in each category. A preliminary dataset was constructed for patient-level separation testing. This “single-image” dataset included images from 120 anemic and 165 non-anemic patients, all with adenomyosis. Because of class imbalance, data from all available anemic patients were included while data from non-anemic patients were selectively sampled. However, results of patient-level evaluation indicated that additional data would be necessary to construct the main dataset. During this process, cases with only low-quality images available, such as those with extreme contrast, were also identified for exclusion. The resulting base dataset comprised 361 images from 276 patients. CBC results obtained within the predefined time window were available; images without corresponding CBC results were excluded from subsequent analyses. In addition, some images from the same patient were dropped to balance the number of images in each class. Overall, 226 images were used for CBC-related datasets.

Three subsets related to CBC were constructed based on these 226 images. The purpose was to assess the separate and combined contributions of image features and CBC data to predictive performance. First, a subset containing feature data from CBC-available patients was generated from the primary dataset and is referred to as “CBC Patient Match” in Table 1. CBC results were then combined with these image features to create the “CBC Data Added” subset. In order to assess the contribution of ultrasound features relative to CBC data, a separate blood test–only dataset was also constructed, indicated as “CBC Data Only”. To summarize, these subsets contain image features, features combined with CBC results, and CBC results only, respectively. It must also be noted that both ROI combinations are also present in the first two subsets.

Although it has been shown that CBC data alone can predict anemia with relatively high accuracy, particularly via hemoglobin levels (Hb) [31], it has been used in this study in combination with image features and uterine size data. Features measured are as the following: Hb, hematocrit (HCT), red blood count (RBC), platelet count (platelet), mean corpuscular volume (MCV), mean corpuscular hemoglobin (MCH), mean corpuscular hemoglobin concentration (MCHC), red cell distribution width (RDW), mean platelet volume (MPV), platelet distribution width (PDW), platelecrit (PCT), prothrombin time (PT sec), international normalized ratio (INR), prothrombin time percent (PT percent), activated partial thromboplastin time (aPTT) [32].

Specific clinical history, such as parity and medical treatment, was inconsistently available, and thus not included in the data. Uterine size measurements, including width and length of the uterine corpus, were added to all datasets except the CBC-only dataset. All primary and derived subsets are summarized in Table 1a,b, while the specific process regarding the addition and elimination of images for each step can be seen in Figure 1.

### 2.3. Preprocessing and Feature Extraction

**Figure 2:**
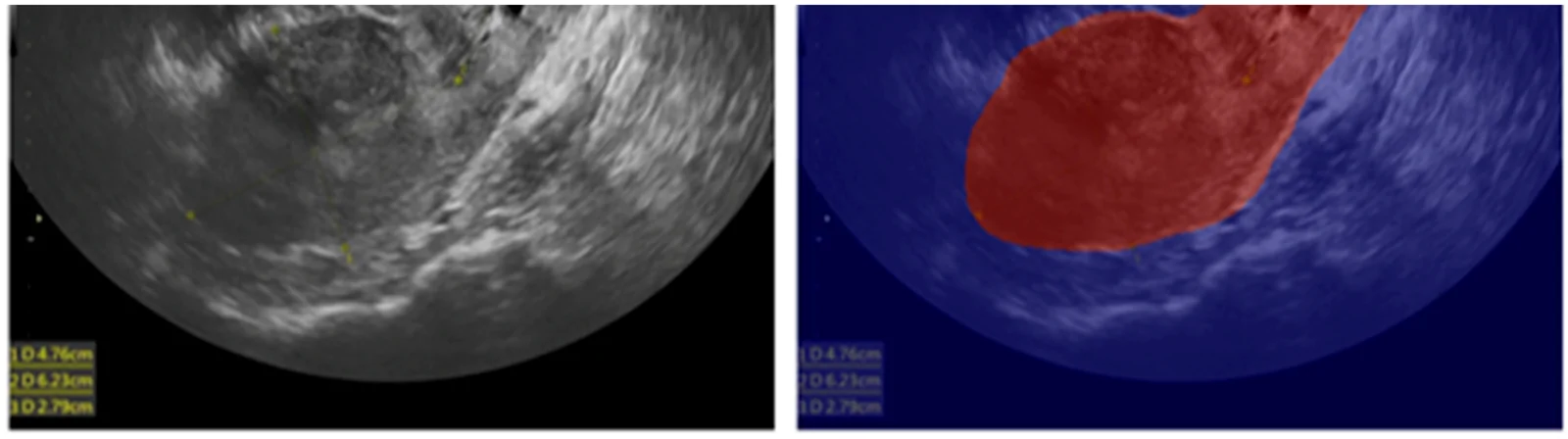
Example of a uterine-specific mask. Colors are added for visibility and were not used during actual processing.

Preprocessing was performed prior to feature extraction for all relevant datasets to minimize fluctuations unrelated to sonographic features. An example can be found below as Figure 2. Elements such as colored markers used for measurements were harmonized with surrounding texture to reduce their impact on radiomic features. Identifying information, including patient IDs and most equipment settings, was removed before full-image masking. Resizing was applied in both ROI categories to mitigate the effects of differing image dimensions, particularly on shape-specific radiomic features. To preserve the original texture as much as possible, each category was standardized to different dimensions. Whole-image ROI data were resized to 875 × 500 pixels (width × height). Uterine-specific ROI images and corresponding masks were resized to 950 × 720 pixels (width × height).

Preliminary tests using conventional deep learning models, such as DINOv2, demonstrated a tendency toward overfitting or poor performance compared with classical machine learning algorithms. Consequently, PyRadiomics, a library designed for extracting quantitative features from medical images, was selected for feature extraction. Microsoft’s Fast and Lightweight AutoML (FLAML) was employed as an auto-optimizing classifier [33,34]. For 2D images, PyRadiomics supports the feature classes listed in Table 2, with the specific number of features for each class based on the official documentation [35]. Classes containing features that were excluded due to redundancy, deprecation, or removal are noted.

### 2.4. Model Training and Statistical Analysis

Figure 1 presents a schematic of the overall study workflow. Nested cross-validation with stratification was used to evaluate the predictive performance of standard machine learning models. Model selection and hyperparameter tuning were automated using FLAML. Validation was performed through a 10-fold outer loop, with the training data in each outer fold undergoing an additional 3- or 5-fold inner cross-validation. The number of inner folds was chosen based on the dataset size to minimize the risk of overfitting. Feature-based evaluation datasets contained 285 and 361 images and underwent 5-fold inner cross-validation. Datasets incorporating CBC data and their corresponding controls contained 226 images. These subsets underwent 3-fold inner cross-validation.

As this study focused on investigating the possibility of radiomics signals related to anemia, rather than evaluating a specific model, model architecture and parameters were optimized independently for each outer loop. The primary decision threshold was set to balance sensitivity and specificity while maintaining predictive power, achieved by maximizing Youden’s J statistic. To assess stability across alternative standards, optimization using the F1-score was also performed with results presented alongside those based on Youden’s J statistic. In addition, fixed-threshold optimization was conducted for the *n* = 361 and *n* = 226 datasets.

Model performance was evaluated using accuracy, sensitivity, specificity, F1-score, and area under the receiver operating characteristic curve (AUC). Except for AUC, all metrics were calculated as follows. TP and FP represent true and false positives, respectively, and TN and FN represent true and false negatives.

(1) $$Accuracy=\frac{TP+TN}{TP+TN+FP+FN}$$

(2) $$Sensitivity=\frac{TP}{TP+FN}$$

(3) $$Specificity=\frac{TN}{TN+FP}$$

(4) $$F1\text{-}Score=\frac{2TP}{2TP+FP+FN}$$

The best-performing models and their corresponding feature importances were documented to identify the most effective discriminative features. Based on preliminary testing, Extreme Gradient Boosting (XGB) and Light Gradient-Boosting Machine (LGBM) were selected for optimization due to their high selection frequency.

## 3. Results

All datasets except the CBC-only included extracted radiomic features with added uterine size information. Images from patients with anemia were labeled as 1 (positive), and non-anemic images were labeled as 0 (negative). Values are reported as mean ± standard deviation (SD) across the 10 outer test folds. AUC is reported as the pooled AUC with 95% bootstrap confidence intervals (CI). In tables, Youden’s J statistic is represented as “Youden’s J”, while F1-score is shortened to “F1” due to space constraints.

### 3.1. Feature-Based Classification

Feature-based datasets were constructed using radiomic image features and uterine size data, with the prevalence of anemic labels at 42% for initial patient-level testing and 54% for all others. These datasets were evaluated to determine whether a relationship could be established between radiomic features and size alone and anemia.

Patient-level feature classification (*N* = 285, *n* = 285) performance was low to modest across all experimental settings. For the whole-image ROI dataset, optimization using Youden’s J statistic yielded an accuracy of 0.592 ± 0.079, with a pooled AUC of 0.570. For the specific ROI dataset, the same Youden’s J statistic optimization produced an accuracy of 0.568 ± 0.094 and a pooled AUC of 0.561. The results, presented in Table 3, suggest limited discriminative performance at the patient level, with AUC values close to 0.5–0.6 and generally low sensitivity.

**Figure 3:**
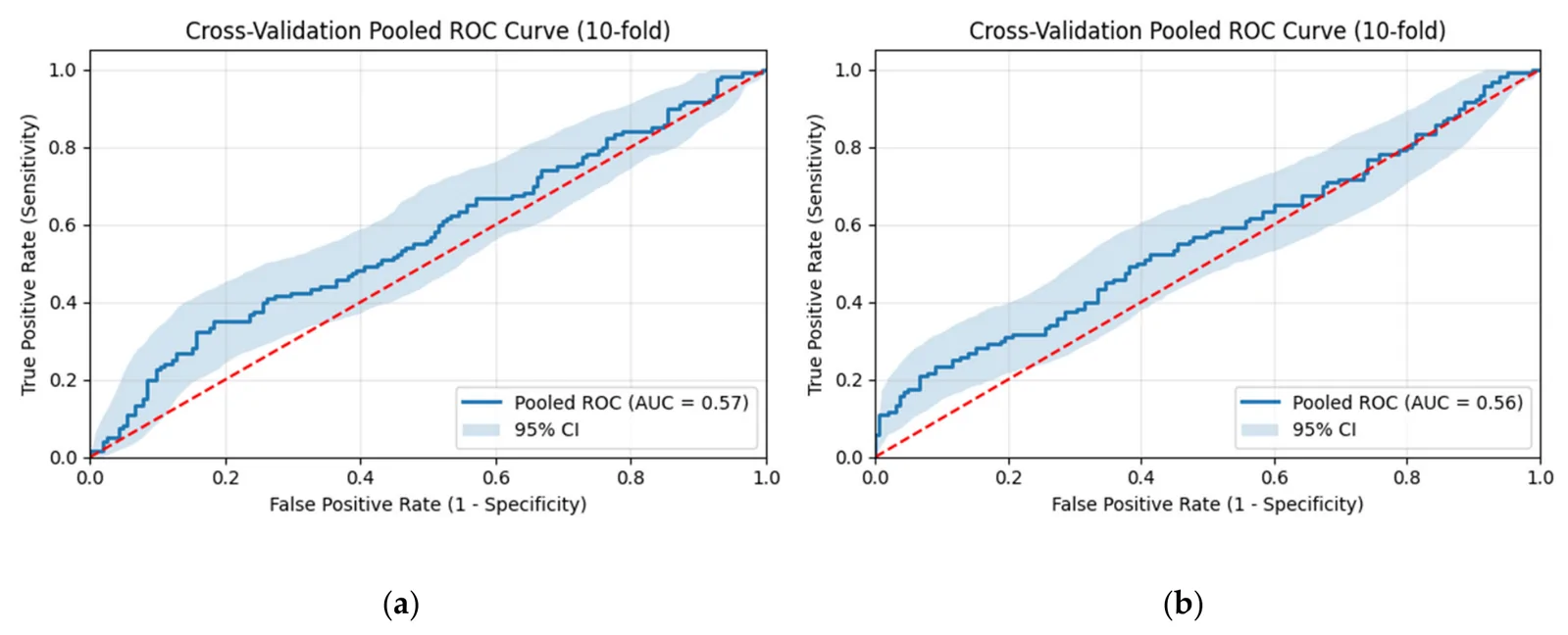
Cross-validated pooled ROC curves for the *n* = 285 feature-based prediction group, for (**a**) specific ROI and (**b**) whole-image ROI. Both were optimized by maximizing Youden’s J statistic.

**Figure A1:**
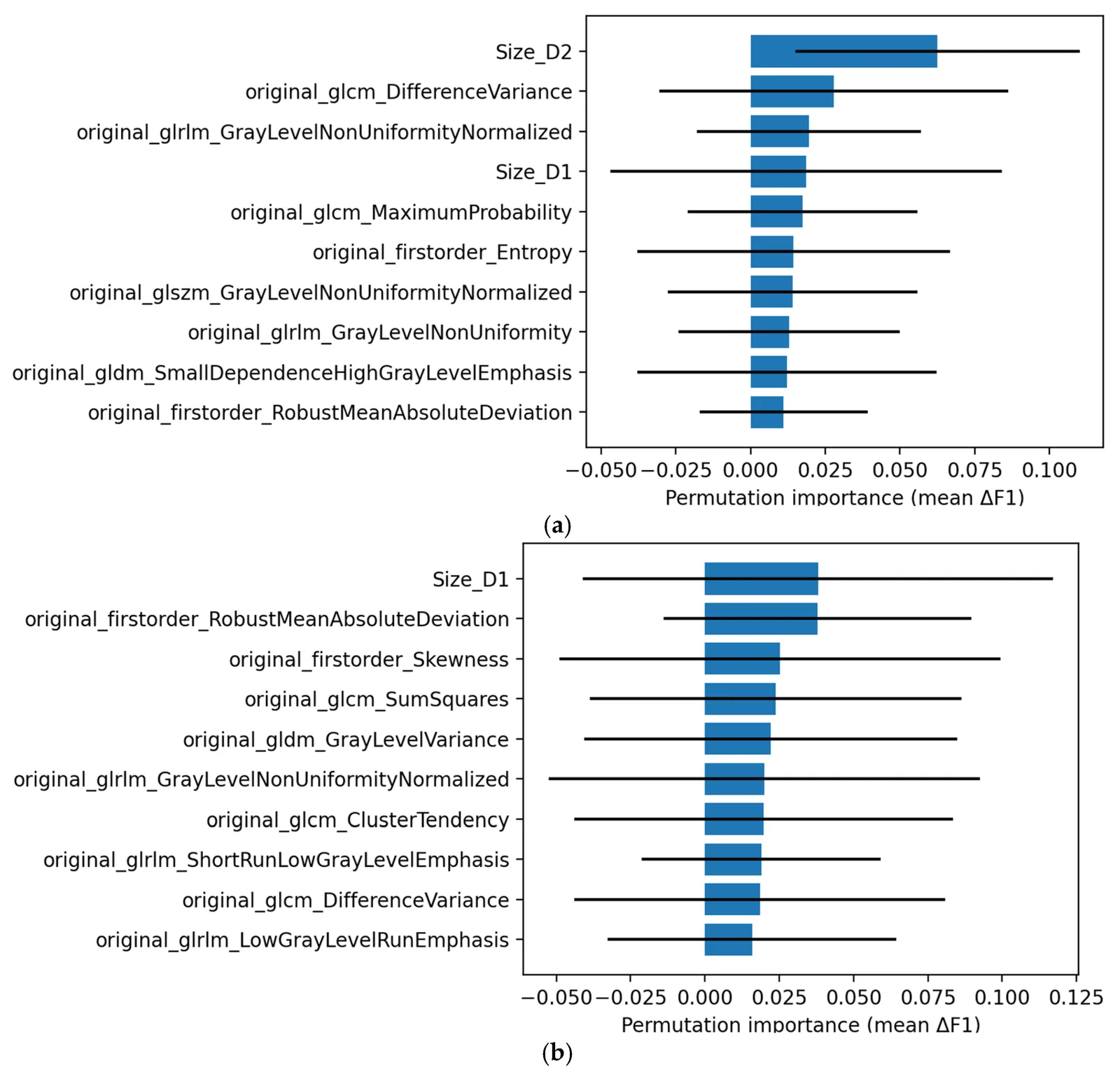
Aggregated permutation importance for the feature-based *n* = 285 prediction group, showing the top 10 ranked features. The thin black line indicates standard deviation. (**a**) Uterine-specific ROI; (**b**) Whole-image ROI. Both were optimized by maximizing Youden’s J statistic.

Notably, this dataset showed a high imbalance between sensitivity and specificity for all approaches. Additional anemic cases and more consistent image-quality control seemed necessary to improve the model’s ability to detect anemia and were subsequently applied to the image selection process for the main feature dataset. The receiver operating characteristic (ROC) curves are demonstrated in Figure 3, while aggregated feature importance can be found in Appendix A, Figure A1 due to space constraints.

**Figure 4:**
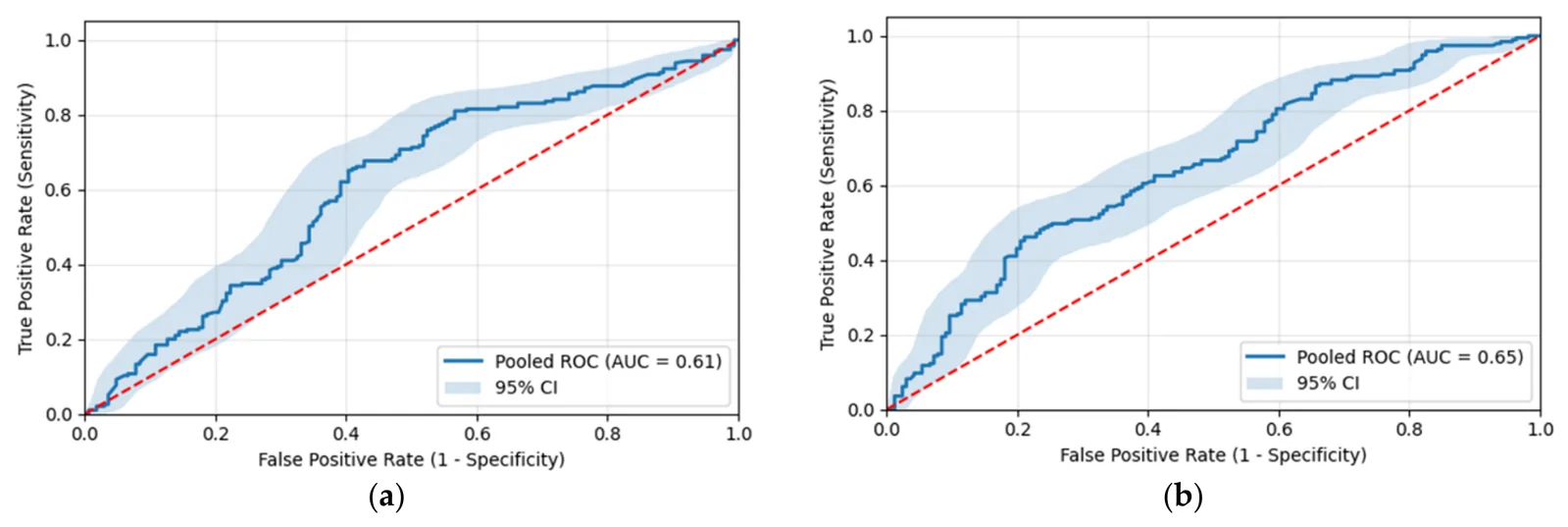
Cross-validated pooled ROC curves for the *n* = 361 feature-based prediction group. (**a**) Specific ROI; (**b**) Whole-image ROI. Both were optimized by maximizing Youden’s J statistic.

Results for the *n* = 361 datasets, without patient-level separation, demonstrated moderate discriminative power for both categories. For the uterine-specific ROI dataset, mean accuracy was 0.618 ± 0.080, with a pooled AUC of 0.612. The whole-image ROI dataset showed a mean accuracy of 0.606 ± 0.080 and a pooled AUC of 0.651. The ROC curves for both can be found below in Figure 4. Overall, uterine-specific ROI data yielded slightly higher accuracy, whereas whole-image ROI data produced a higher overall AUC. Because AUC reflects performance across all possible thresholds, this discrepancy may indicate a substantial number of intermediate cases that were difficult to classify correctly using the methods applied.

Accuracy, pooled AUC, and model selection frequency showed minimal differences between ROI categories. Sensitivity and specificity exhibited more notable variation, likely due to the 8% class imbalance, with a higher number of anemic images biasing predictions toward positive labels. This resulted in higher sensitivity relative to specificity. Other metrics remained largely consistent across decision thresholds, suggesting that while discriminative signals were modest, they were relatively robust to threshold selection. Thus, predictive performance remained stable. Specific metrics are summarized in Table 4. Additional results for fixed-threshold optimization are presented in Appendix B, Table A1.

**Figure 5:**
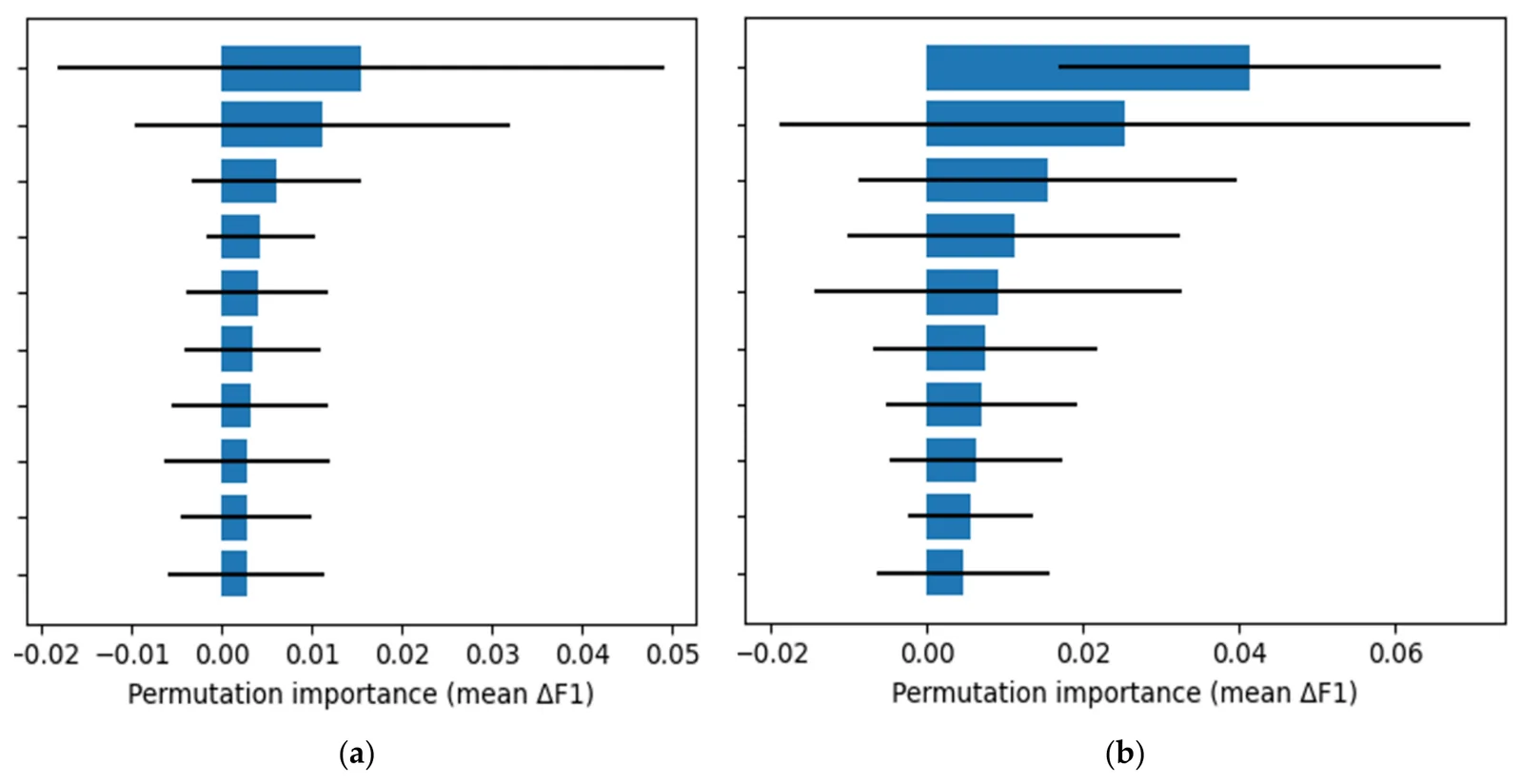
Aggregated permutation importance for the feature-based *n* = 361 prediction group, showing the top 10 ranked features. The thin black line indicates standard deviation. As stated in the text, feature names have been moved to the Appendix A due to space con-straints. (**a**) Uterine-specific ROI; (**b**) Whole-image ROI. Optimization standards are the same as in Figure 3 and Figure 4.

The two ROI categories shared very few high-ranking features in terms of feature importance. The absence of Shape_2D features among top-ranking features in the whole-image ROI dataset is likely due to size standardization. In contrast, the uterine-specific ROI dataset exhibited mostly similar feature importance values, whereas the whole-image ROI dataset showed more pronounced differences among top-ranking features. These are illustrated in Figure 5. Due to space constraints, however, specific feature names have been moved to Appendix B Table A2.

Table 5 presents results for non-resized uterine-specific ROI features. Accuracy was lower than that observed for the resized dataset (0.618 ± 0.080), while the AUC was slightly higher at 0.645. Expanding on previous observations, it is likely that intermediate cases limiting classification accuracy were more prevalent in the non-resized configuration, possibly due to variable image dimensions. Although resizing may alter image texture, in this study the overall effect on performance metrics appeared favorable.

Because CBC data were unavailable for some patients, a subset of the feature-based dataset was constructed to allow a fair comparison between image features and blood test data. This subset included 226 images from the original 361, with anemic and non-anemic cases equally represented (1:1 ratio). The “CBC Patient Match” dataset contains configurations derived from the same images as the “CBC Data Added” datasets described later, but includes only extracted radiomic features and size data. This subset was necessary to evaluate the impact of reduced sample size and to provide a baseline for comparison with CBC-augmented datasets. Relevant metrics are summarized in Table 6.

ROI category appeared to significantly influence predictive performance in this group. For uterine-specific ROI datasets, overall performance was low: accuracy and F1-score were comparable to random classification. In contrast, whole-image ROI datasets exhibited improved performance across nearly all metrics. The exception was sensitivity and specificity, with higher specificity indicating that the model tended to avoid false positives. This may be attributable to the smaller dataset size and correspondingly lower numbers of samples per fold.

**Figure 6:**
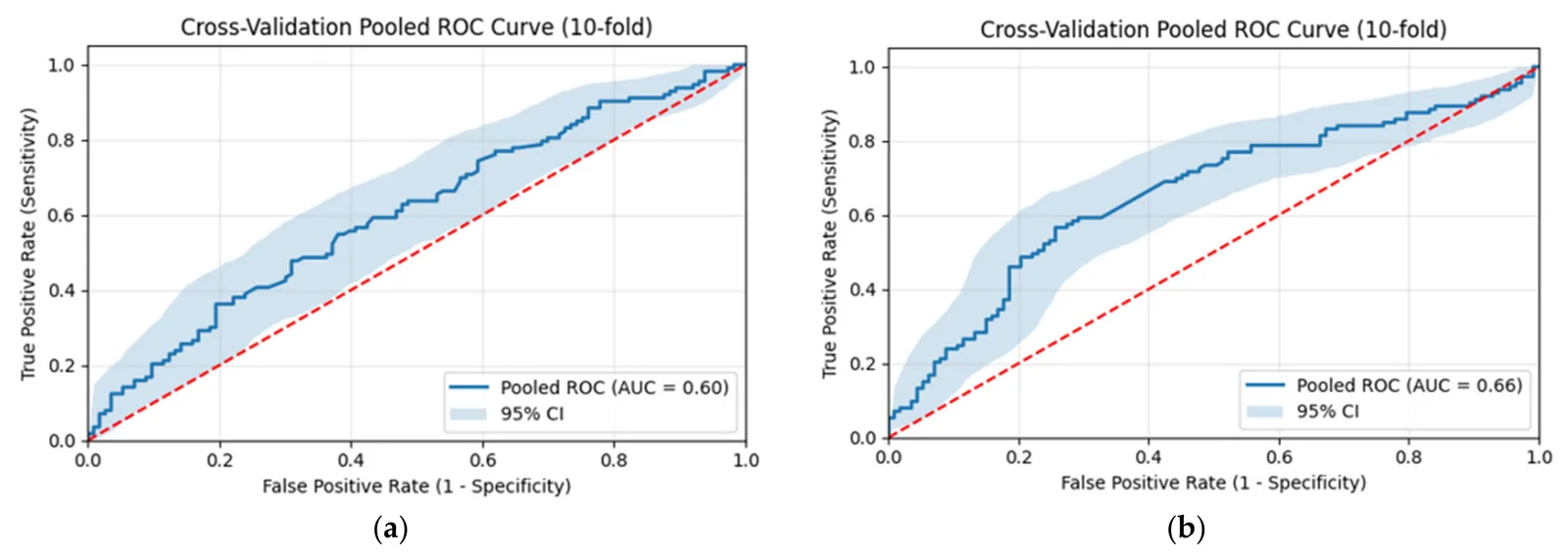
ROC curve comparison. (**a**) Uterine-specific ROI; (**b**) Whole-image ROI. Both were optimized by maximizing Youden’s J statistic.

Figure 6 presents the ROC curves for each ROI category. Compared with the original dataset, uterine-specific ROI data showed a substantial decrease in performance, suggesting that reduced sample size can destabilize feature-driven classification. The observed increase in performance, coupled with imbalance between sensitivity and specificity, may also stem from the same cause. However, the consistently higher AUC for whole-image ROI datasets suggests that potential anemia-related markers are not limited to the uterine corpus. Restricting the ROI to a specific region may exclude features that, while subtle, carry predictive significance.

### 3.2. CBC Data and Feature-Based Classification

Datasets including CBC variables were analyzed to evaluate classification performance. Three subsets were tested using 10-fold nested cross-validation: image feature datasets for each ROI category with added CBC data, and a CBC-only dataset. Results for Youden’s J statistic and F1-score optimization are summarized in Table 7. Additional results for fixed-threshold optimization are presented in Appendix B, Table A1.

**Figure 7:**
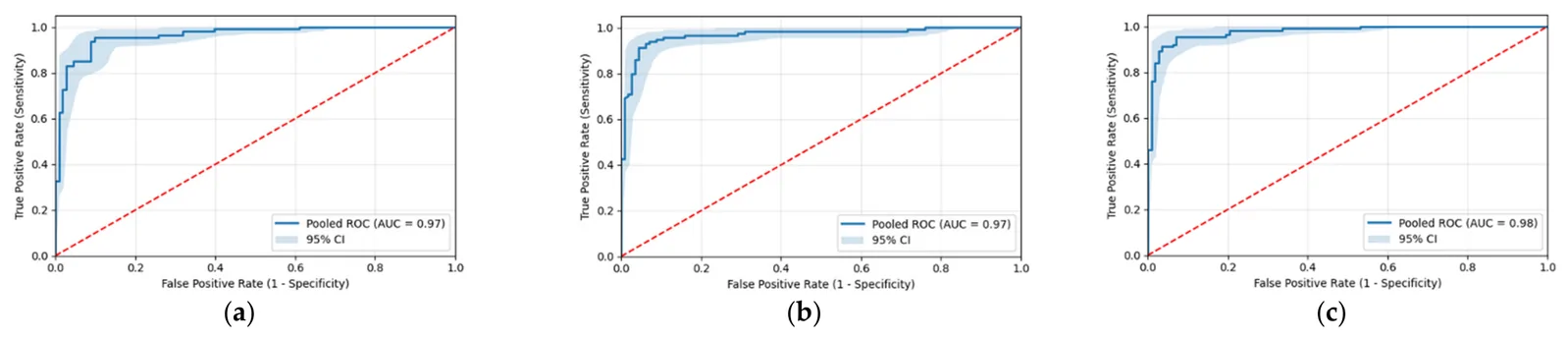
ROC curve comparison for datasets with CBC data. From left to right: (**a**) Uterine-specific ROI with CBC data; (**b**) Whole-image ROI with CBC data; (**c**) CBC data only.

Notably, the CBC-only dataset slightly outperformed the others in terms of AUC, which can also be seen in the ROC curve comparison in Figure 7. This suggests that the extracted radiomic features did not provide additional predictive value beyond what was obtainable from CBC data alone. Because testing conditions were consistent across all datasets, results for maximum F1-score and Youden’s J statistic were identical for all three sets. This reflects the stability of the classification, indicating that the data were relatively easy to classify.

**Figure 8:**
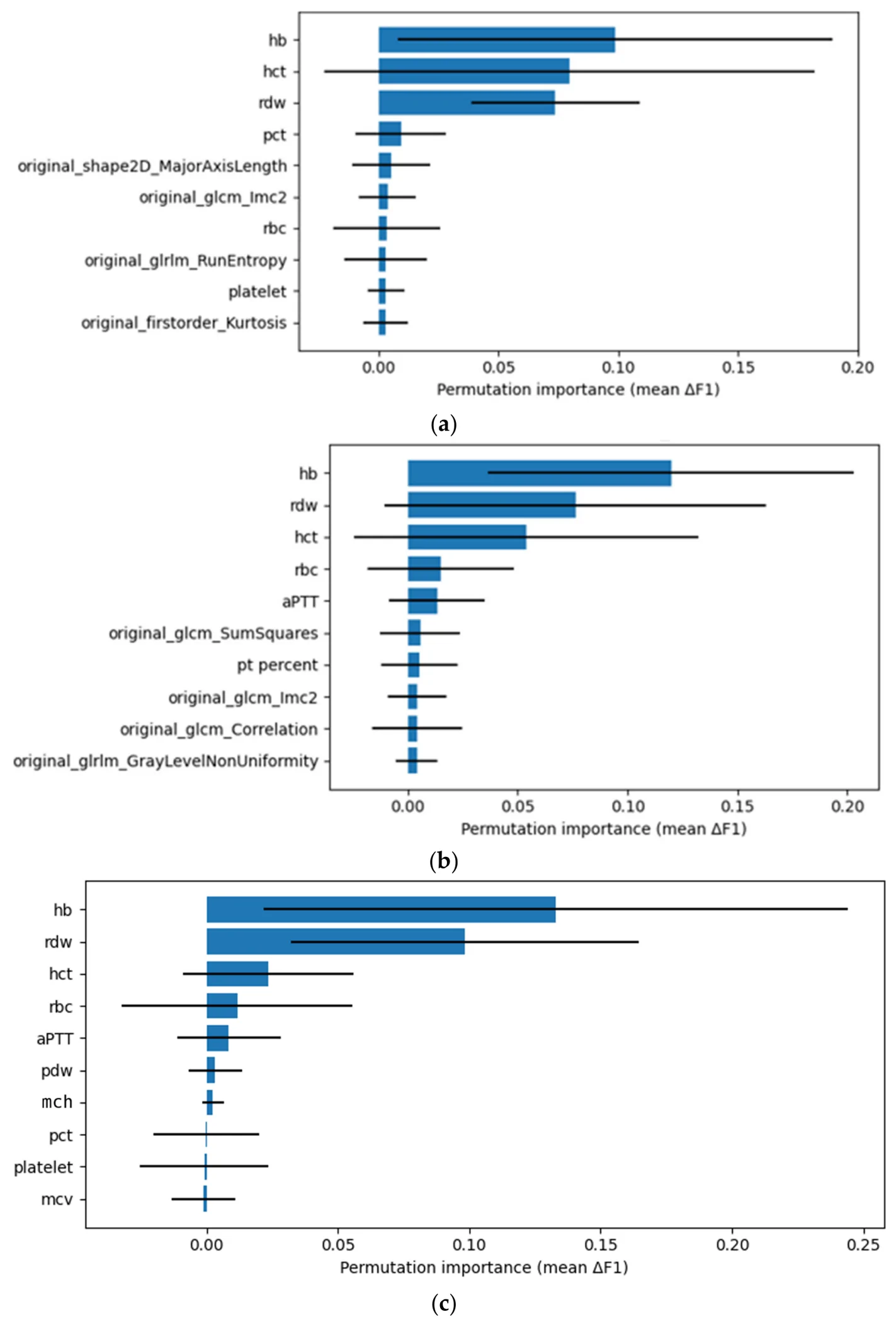
Feature importance for CBC datasets. (**a**) Uterine ROI with CBC data; (**b**) Whole-image ROI with CBC data; (**c**) CBC data only. Grid sizes differ across panels.

As illustrated in Figure 8, most of the features with the highest permutation importance were CBC variables. Hb, RDW, and HCT, which are well-established indicators of anemia, consistently occupied the top three positions. Some differences were observed in terms of which radiomic features, along with some CBC variables, retained high importance between the two ROI datasets. A notable example is aPTT, a measure of the time required for clot formation. While aPTT contributed meaningfully to classification in the whole-image ROI and CBC-only datasets, it was absent from the uterine-specific ROI permutation importance rankings. Overall, CBC data substantially enhanced classification precision. However, the relatively large difference between accuracy and AUC indicates the presence of borderline cases that were more difficult to classify.

## 4. Discussion

This study evaluated whether quantitative radiomic features extracted from B-mode greyscale TVUS images could be used to detect anemia in patients with adenomyosis. The main findings can be summarized as follows: (1) extracted radiomic features demonstrated moderate discriminative capability, and (2) performance was strongly influenced by preprocessing. Additionally, while combining CBC data with radiomic features improved predictive power, CBC data alone exhibited a higher capacity for detecting anemia.

The consistently lower sensitivity relative to specificity for single-image tests may reflect limitations of the dataset. High variability in sensitivity across folds was also observed, suggesting unstable positive-class representation during cross-validation. Therefore, these results were interpreted as an indication that the dataset may be underpowered or insufficiently balanced for reliable detection of anemic patients. In this case, a possible factor was the relatively small number of anemic cases as well as overall images, and subsequently more data was added to construct the *n* = 361 dataset for further feature evaluation.

Across further feature-based configurations—including radiomic features and uterine size data—both ROI categories showed some discriminative capability. Uterine-specific ROIs achieved slightly higher accuracy, whereas whole-image ROIs consistently demonstrated higher pooled AUC. Changing the threshold optimization strategy between maximizing Youden’s J statistic and F1-score primarily affected sensitivity and specificity, with minimal impact on accuracy and pooled AUC. This pattern suggests that several cases lie near the classification threshold, making them difficult to categorize correctly. The limited effect of thresholding indicates that these classification challenges are more likely due to weak signal intensity rather than threshold selection.

The impact of image preprocessing is evident in the differing performances of the two ROI categories. While overall accuracy, particularly for feature-based classification, remained similar, feature importance rankings showed minimal overlap. For uterine-specific ROIs, shape-related features tended to rank higher, whereas size features were more prominent in whole-image ROIs. Both ROI categories were influenced by texture features, albeit in different classes. Differences in ROI selection determine which features are included during extraction. Uterine-specific ROIs capture shape information, which serves as a distinctive feature, whereas whole-image masks encompass broader texture and contextual information both within and outside the uterine region, including changes at the margins. These findings indicate that anemia-related radiomic features may be diffusely distributed across uterine tissue rather than concentrated in a focal region.

However, caution is warranted in interpreting these results. Feature differences may arise not only from the uterus but also from contextual artifacts unrelated to adenomyosis, such as background texture or variations in acquisition methods. This concern is particularly relevant for whole-image ROIs, which include a larger area during feature extraction.

Size standardization also improved performance in certain configurations. For whole-image ROIs, standardization rendered shape-related features largely redundant, explaining the observed differences in feature importance. While resizing can potentially alter image texture, the target dimensions were chosen to minimize required transformations. In future iterations, adapting to variations in physical pixel spacing may further optimize results.

Adding CBC data to the datasets substantially increased predictive power; however, performance remained lower than when using CBC data alone. The dominant influence of CBC variables was reflected in feature importance rankings: hemoglobin (Hb), red cell distribution width (RDW), and hematocrit (HCT) consistently occupied the top three positions across all datasets. These findings indicate that CBC data is more informative than radiomics features in this context, but image-derived features may provide supplementary value in situations where complete CBC data are unavailable, such as delays or missing records. Approximately 30% of the total feature importance in the CBC-only dataset was attributed to these three key variables. When one or more of these variables are missing, radiomics features could still contribute relevant information for anemia prediction.

Finally, subsets restricted to patients with CBC data exhibited different patterns compared with the larger datasets. Model selection was broadened to include algorithms such as Random Forest, resulting in greater variability in performance metrics. In some cases, accuracy and pooled AUC improved, but the gap between sensitivity and specificity also increased. These effects were likely driven by the smaller sample size and the resulting increase in model instability. It is also important to note that these subsets were limited to patients with available CBC results; unmeasured factors influencing data availability—such as symptom severity—may have affected classification outcomes.

While this study provides preliminary evidence that routine TVUS images of adenomyosis patients contain information related to anemia status, several limitations must be acknowledged. First, although the original images clearly displayed features of adenomyosis, 2D greyscale still frames may contain less information than alternative modalities, such as 3D transvaginal ultrasound (TVUS). These were considered during study planning but ultimately excluded due to even more limited data availability. Clinical deployment would require greater robustness, which remains to be addressed in future work, as this study was intended as a proof-of-concept. Another limitation was the availability of CBC data. The collection window was set to six months to maximize data inclusion; however, this represents a relatively large interval, and future studies may benefit from narrower time constraints. Additionally, the images may be affected by operator-dependent variability and vendor-specific imaging characteristics. For instance, some images exhibited a distinctive blue hue depending on the ultrasound machine, which was corrected during preprocessing. More subtle signatures may have persisted and thereby influenced feature extraction. The main feature dataset included multiple images per patient. Although this improved the balance between sensitivity and specificity, patient-level grouping was not enforced during 10-fold nested cross-validation for this dataset. Images from the same patient could therefore have been assigned to both training and test folds, introducing a risk of data leakage and potentially inflating performance estimates. Future studies should enforce patient-level separation at every stage of model development or employ architectures that process one image per patient through separate model branches. In order to achieve both a balanced dataset and prevention of data leakage, clinically appropriate data augmentation strategies should be considered for subsequent studies. External validation using images acquired at other institutions and with different ultrasound systems may also be necessary to assess generalizability. Finally, implementing automated uterine segmentation could improve efficiency and reproducibility.

## 5. Conclusions

This proof-of-concept study evaluated whether radiomics features extracted from B-mode greyscale sonographic images were associated with anemia status in adenomyosis patients. Two regions of interest (ROIs) were analyzed: one encompassing the uterine corpus and the other including the entire image. In the preliminary patient-level single-image analysis, image-derived features showed only low-to-modest discrimination, indicating limited reliability for stand-alone anemia prediction. An expanded image dataset was utilized to address the initial imbalance in sensitivity and specificity in the single-image dataset, yielding modest predictive performance with accuracy around 0.61 across both ROIs. Preprocessing and ROI selection were shown to significantly influence all performance metrics, likely reflecting the varying contextual information captured within the same image data.

Combining radiomics features with CBC data improved predictive power; however, CBC-only datasets performed slightly better than the combined datasets. This suggests that, when highly discriminatory clinical variables are available, radiomics-derived image features do not provide clear incremental value. Overall, routine TVUS images may contain subtle complementary information related to anemia status in patients with adenomyosis. At this stage, radiomics features should therefore be regarded as supplementary, with potential clinical utility primarily in settings where blood test results are incomplete or unavailable. Future studies should apply stricter temporal constraints for CBC data collection and enforce patient-level separation throughout training, validation, and testing to minimize potential data leakage. Larger, balanced cohorts are also needed to improve model stability and permit more reliable estimation, possibly with clinically appropriate data augmentation. External validation using datasets from other centers or scanners is also recommended to assess generalizability.

## Figures and Tables

**Table 1:** Patient demographics and data configuration. (**a**) Feature-based datasets; (**b**) subsets related to CBC data.

(**a**)			
	Single Image		Original Image Features
ROI/analysis type	Specific ROI; Whole ROI		Specific ROI; Whole ROI
Input features	Radiomics + uterine size		Radiomics + uterine size
Image/case count (*n*)	285		361
Patient count (*N*)	285		276
Age range (years)	28–50		28–50
Age, mean ± SD	45.49 ± 3.79		45.46 ± 3.79
Age, median	46		46
Anemic cases (%)	42%		54%
(**b**)			
	CBC Patient Match	CBC Data Added	CBC Data Only
ROI/analysis type	Specific ROI; Whole ROI	Specific ROI; Whole ROI	CBC data only
Input features	Radiomics + uterine size	Radiomics + uterine size + CBC data	CBC data only
Image/case count (*n*)	226	226	226
Patient count (*N*)	175	175	175
Age range (years)	28–50	28–50	28–50
Age, mean ± SD	45.38 ± 3.90	45.38 ± 3.90	45.38 ± 3.90
Age, median	46	46	46
Anemic cases (%)	50%	50%	50%

Note: Abbreviations: ROI as described in the text; SD: Standard Deviation.

**Table 2:** PyRadiomics supported feature classes for 2D images.

Class Name	Abbreviation	Number of Features
First Order Statistics	firstorder	19 *
Shape-Based (2D)	shape2D	16 †
Gray Level Co-occurrence Matrix	glcm	24 †
Gray Level Run Length Matrix	glrlm	16
Gray Level Size Zone Matrix	glszm	16
Neighboring Gray Tone Difference Matrix	ngtdm	5
Gray Level Dependence Matrix	gldm	14
Total number of features: Total number of features used in extraction:		110 104

* The feature “firstorder_StandardDeviation” was removed because it is redundant with “firstorder_Variance”, standard deviation being the square root of variance. † Includes deprecated or removed features.

**Table 3:** Feature-based prediction on *n* = 285 dataset.

Dataset	Optimization	Accuracy	F1-Score	Sensitivity	Specificity	Pooled AUC (95% CI)	Model Selection
Specific ROI	Youden’s J	0.568 ± 0.094	0.419± 0.138	0.383 ± 0.153	0.701 ± 0.151	0.561 (0.490, 0.627)	LGBM: 5/10 XGB: 2/10 LRL2: 2/10 LRL1: 1/10
	F1	0.582 ± 0.091	0.474 ± 0.158	0.475 ± 0.204	0.658 ± 0.136	0.565 (0.492, 0.635)	LGBM: 3/10 XGB: 2/10 LRL2: 3/10 LRL1: 2/10
Whole-Image ROI	Youden’s J	0.592 ± 0.079	0.356 ± 0.244	0.342 ± 0.285	0.777 ± 0.140	0.570 (0.500, 0.637)	LGBM: 6/10 XGB: 3/10 RF: 1/10
	F1	0.600 ± 0.064	0.344 ± 0.254	0.333 ± 0.283	0.796 ± 0.175	0.604 (0.534, 0.668)	LGBM: 7/10 XGB: 2/10 RF: 1/10

Note: Abbreviations: LGBM, XGB as described in the text; LRL2: Ridge-Penalized Linear Regression; LRL1: Lasso-Penalized Linear Regression [36]; RF: Random Forest.

**Table 4:** Feature-based prediction on *n* = 361 dataset.

Dataset	Optimization	Accuracy	F1-Score	Sensitivity	Specificity	Pooled AUC (95% CI)	Model Selection
Specific ROI	Youden’s J	0.618 ± 0.080	0.650 ± 0.090	0.669 ± 0.133	0.554 ± 0.156	0.612 (0.554, 0.672)	LGBM: 6/10 XGB: 4/10
	F1	0.610 ± 0.100	0.671 ± 0.094	0.747 ± 0.138	0.444 ± 0.201	0.612 (0.553, 0.671)	LGBM: 6/10 XGB: 4/10
Whole-Image ROI	Youden’s J	0.606 ± 0.080	0.601 ± 0.107	0.568 ± 0.151	0.651 ± 0.111	0.651 (0.590, 0.707)	LGBM: 5/10 XGB: 5/10
	F1	0.612 ± 0.086	0.645 ± 0.087	0.661 ± 0.117	0.555 ± 0.120	0.641 (0.580, 0.699)	LGBM: 6/10 XGB: 4/10

Note: Abbreviations: LGBM, XGB as described in the text.

**Table 5:** 10-fold nested CV results for non-standardized image size, uterine-specific ROI dataset (*n* = 361).

Dataset	Optimization	Accuracy	F1-Score	Sensitivity	Specificity	Pooled AUC (95% CI)	Model Selection
Specific ROI, Non-Standardized	Youden’s J	0.585 ± 0.072	0.599 ± 0.067	0.586 ± 0.133	0.585 ± 0.215	0.645 (0.587, 0.703)	LGBM: 7/10 XGB: 3/10
	F1	0.635 ± 0.060	0.704 ± 0.052	0.810 ± 0.098	0.427 ± 0.126	0.645 (0.587, 0.703)	LGBM: 7/10 XGB: 3/10

Note: Abbreviations: LGBM, XGB as described in the text.

**Table 6:** Feature-based prediction on the “CBC Patient Match” dataset (*n* = 226).

Dataset	Optimization	Accuracy	F1-Score	Sensitivity	Specificity	Pooled AUC (95% CI)	Model Selection
Specific ROI	Youden’s J	0.554 ± 0.067	0.543 ± 0.153	0.583 ± 0.211	0.527 ± 0.211	0.605 (0.532, 0.674)	LGBM: 5/10 XGB: 4/10 LRL2: 1/10
	F1	0.549 ± 0.072	0.541 ± 0.155	0.583 ± 0.211	0.518 ± 0.208	0.605 (0.532, 0.674)	LGBM: 5/10 XGB: 4/10 LRL2: 1/10
Whole-Image ROI	Youden’s J	0.636 ± 0.096	0.520 ± 0.221	0.448 ± 0.218	0.823 ± 0.145	0.657 (0.582, 0.727)	XGB: 4/10 LGBM: 4/10 RF: 1/10 XT: 1/10
	F1	0.632 ± 0.108	0.610 ± 0.121	0.594 ± 0.193	0.673 ± 0.275	0.657 (0.582, 0.727)	XGB: 4/10 LGBM: 4/10 RF: 1/10 XT: 1/10

Note: Abbreviations: LGBM, XGB as described in the text; LRL2: Ridge-Penalized Linear Regression; RF: Random Forest; XT: Extremely Randomized Trees (commonly denoted as “extra_tree”).

**Table 7:** Classification results for datasets with added CBC data (*n* = 226).

Dataset	Optimization	Accuracy	F1-Score	Sensitivity	Specificity	Pooled AUC (95% CI)	Model Selection
Specific ROI	Youden’s J	0.889 ± 0.085	0.871 ± 0.112	0.807 ± 0.165	0.973 ± 0.043	0.966 (0.942, 0.985)	LGBM: 6/10 XGB: 4/10
	F1	0.889 ± 0.085	0.871 ± 0.112	0.807 ± 0.165	0.973 ± 0.043	0.966 (0.942, 0.985)	LGBM: 6/10 XGB: 4/10
Whole-Image ROI	Youden’s J	0.890 ± 0.075	0.879 ± 0.081	0.823 ± 0.126	0.958 ± 0.106	0.967 (0.943, 0.988)	XGB: 6/10 LGBM: 4/10
	F1	0.890 ± 0.075	0.879 ± 0.081	0.823 ± 0.126	0.958 ± 0.106	0.967 (0.943, 0.988)	XGB: 6/10 LGBM: 4/10
CBC Data Only	Youden’s J	0.930 ± 0.042	0.926 ± 0.045	0.894 ± 0.071	0.964 ± 0.046	0.978 (0.961, 0.992)	LGBM: 5/10 XT: 3/10 XGB: 2/10
	F1	0.930 ± 0.042	0.926 ± 0.045	0.894 ± 0.071	0.964 ± 0.046	0.978 (0.961, 0.992)	LGBM: 5/10 XT: 3/10 XGB: 2/10

Note: Abbreviations: LGBM, XGB as described in the text; XT: Extremely Randomized Trees.

## Data Availability

The data used contain sensitive clinical information and will be shared upon reasonable request, after appropriate review and approval.

## Abbreviations

The following abbreviations are used in this manuscript:

AUB	Abnormal Uterine Bleeding
CBC	Complete Blood Count
SD	Standard Deviation
HCT	Hematocrit
Hb	Hemoglobin
RDW	Red Cell Distribution Width
ROI	Region of Interest
TVUS	Transvaginal Ultrasound
PyRadiomics	Python Radiomics
FLAML	Fast and Lightweight AutoML
XGB	Extreme Gradient Boosting
LGBM	Light Gradient-Boosting Machine
CI	Confidence Interval
Youden’s J	Youden’s J statistic
F1	F1-score
RF	Random Forest
XT	Extremely Randomized Trees
LRL1	Logistic Regression with L1 regularization (Lasso Penalized Linear Regression)
LRL2	Logistic Regression with L2 regularization (Ridge Penalized Linear Regression)
AUC	Area Under the Receiver-Operating Characteristic Curve
TP	True Positive
TN	True Negative
FP	False Positive
FN	False Negative
CV	Cross-Validation
nnU-net	no new U-net
aPTT	Activated Partial Thromboplastin Time

## Appendix B

**Table A1:** Summary of 10-fold nested CV, threshold = 0.5.

Configuration	Image Number (*n*)	ROI and Preprocessing	Feature Set	Accuracy	F1	Sen.	Spec.	Pooled AUC (95% CI)	Selected Models
Original Image Features	361	Corpus ROI	Radiomics + uterine size	0.607 ± 0.055	0.702 ± 0.043	0.862 ± 0.084	0.308 ± 0.120	0.624 (0.564–0.681)	XGBoost 7, LightGBM 3
Original Image Features, Whole Resized	361	Whole-image mask + resizing	Radiomics + uterine size	0.543 ± 0.032	0.678 ± 0.043	0.903 ± 0.130	0.122 ± 0.131	0.602 (0.543–0.659)	LightGBM 5, XGBoost 5
CBC Patient Match	226	Corpus ROI	Radiomics + uterine size	0.518 ± 0.057	0.538 ± 0.103	0.595 ± 0.217	0.445 ± 0.206	0.530 (0.453–0.604)	XGBoost 8, ExtraTrees 1, RF 1
CBC Patient Match, Whole Resized	226	Whole-image mask + resizing	Radiomics + uterine size	0.650 ± 0.126	0.595 ± 0.240	0.590 ± 0.253	0.712 ± 0.204	0.657 (0.584–0.729)	LightGBM 5, XGBoost 4, RF 1
CBC Data Added	226	Corpus ROI	Radiomics + uterine size + CBC	0.920 ± 0.045	0.923 ± 0.040	0.938 ± 0.043	0.902 ± 0.089	0.966 (0.943–0.985)	XGBoost 5, LightGBM 3, ExtraTrees 2
CBC Data Added, Whole Resized	226	Whole-image mask + resizing	Radiomics + uterine size + CBC	0.930 ± 0.077	0.933 ± 0.071	0.946 ± 0.063	0.913 ± 0.125	0.968 (0.944–0.988)	XGBoost 7, LightGBM 3
CBC Data Only	226	N/A	CBC only	0.939 ± 0.051	0.940 ± 0.048	0.939 ± 0.042	0.938 ± 0.084	0.980 (0.964–0.993)	LightGBM 4, ExtraTrees 3, XGBoost 3

Note: Mean ± SD are across the 10 outer test folds; AUC is reported as pooled AUC with 95% bootstrap confidence interval.

**Table A2:** Top 10 features for feature-based prediction using specific and whole ROIs.

Uterine ROI	Whole-Image ROI
original_shape2D_MinorAxisLength	Size_D1
original_shape2D_Sphericity	original_glszm_SmallAreaHighGrayLevelEmphasis
original_glrlm_LowGrayLevelRunEmphasis	original_firstorder_Entropy
original_glszm_LargeAreaLowGrayLevelEmphasis	original_glrlm_GrayLevelNonUniformity
original_glszm_SizeZoneNonUniformityNormalized	Size_D2
original_gldm_DependenceVariance	original_firstorder_Kurtosis
original_gldm_LargeDependenceHighGrayLevelEmphasis	original_firstorder_Variance
original_firstorder_Skewness	original_glcm_MaximumProbability
original_glszm_GrayLevelNonUniformity	original_glszm_GrayLevelVariance
